# Plasma Metabolic Disturbances in Parkinson’s Disease Patients

**DOI:** 10.3390/biomedicines10123005

**Published:** 2022-11-22

**Authors:** Paulina Gątarek, Joanna Sekulska-Nalewajko, Barbara Bobrowska-Korczaka, Małgorzata Pawełczyk, Karol Jastrzębski, Andrzej Głąbiński, Joanna Kałużna-Czaplińska

**Affiliations:** 1 Institute of General and Ecological Chemistry, Faculty of Chemistry, Lodz University of Technology, 90-924 Lodz, Poland; 2 CONEM Poland Chemistry and Nutrition Research Group, Lodz University of Technology, 90-924 Lodz, Poland; 3 Institute of Applied Computer Science, Lodz University of Technology, 90-924 Lodz, Poland; 4 Department of Bromatology, Faculty of Pharmacy, Medical University of Warsaw, 02-097 Warsaw, Poland; 5 Department of Neurology and Stroke, Medical University of Lodz, 90-549 Lodz, Poland

**Keywords:** biomarkers, Parkinson’s disease, PD, plasma, metabolomics, metabolites, GC-TOFMS, chromatographic techniques

## Abstract

Plasma from patients with Parkinson’s disease (PD) is a valuable source of information indicating altered metabolites associated with the risk or progression of the disease. Neurotoxicity of dopaminergic neurons, which is triggered by aggregation of α-synuclein, is the main pathogenic feature of PD. However, a growing body of scientific reports indicates that metabolic changes may precede and directly contribute to neurodegeneration. Identification and characterization of the abnormal metabolic pattern in patients’ plasma are therefore crucial for the search for potential PD biomarkers. The aims of the present study were (1) to identify metabolic alterations in plasma metabolome in subjects with PD as compared with the controls; (2) to find new potential markers, some correlations among them; (3) to identify metabolic pathways relevant to the pathophysiology of PD. Plasma samples from patients with PD (*n* = 25) and control group (*n* = 12) were collected and the gas chromatography-time-of-flight-mass spectrometry GC-TOFMS-based metabolomics approach was used to evaluate the metabolic changes based on the identified 14 metabolites with significantly altered levels using univariate and multivariate statistical analysis. The panel, including 6 metabolites (L-3-methoxytyrosine, aconitic acid, L-methionine, 13-docosenamide, hippuric acid, 9,12-octadecadienoic acid), was identified to discriminate PD from controls with the area under the curve (AUC) of 0.975, with an accuracy of 92%. We also used statistical criteria to identify the significantly altered level of metabolites. The metabolic pathways involved were associated with linoleic acid metabolism, mitochondrial electron transport chain, glycerolipid metabolism, and bile acid biosynthesis. These abnormal metabolic changes in the plasma of patients with PD were mainly related to the amino acid metabolism, TCA cycle metabolism, and mitochondrial function.

## 1. Introduction

Parkinson’s disease (PD) is a neurodegenerative disease of the central nervous system (CNS) characterized by a progressive loss of dopaminergic neurons in the substantia nigra and the production of Lewy bodies [1,2,3]. Dopamine-producing neurons (DPNs) are prone to degeneration due to the extensive branching and a significant amount of energy required to transmit nerve signals along this network. DPNs are pacemaking neurons, which means they are continuously discharged. Therefore, they need a lot of energy to be recharged. The degeneration process occurs when they run out of energy [4]. Loss of dopamine neurons causes progressive impairment of motor control, which is the primary clinical feature of this disease. Typical motor symptoms appear when more than 80% of dopaminergic cells are lost. The diagnosis of PD is most often made on the basis of the presence of characteristic PD motor symptoms. Motor symptoms include rigidity of muscles, bradykinesia, resting tremor, postural deformities, and problems with balance. Due to progressive neurodegeneration, patients with PD also have non-motor symptoms such as sensory abnormalities, sleep disturbances, autonomic dysfunction, behavioral changes, dementia, depression, psychosis, anxiety, and fatigue. Treatment of PD is mainly based on symptomatic treatment to reduce the severity of symptoms. Unfortunately, this treatment does not stop nor slow down the progression of neuronal degeneration [3]. There are a lot of theories about the specific causes of PD [1,2]. To date, the etiology of PD is still not well understood. Researchers point out that all sorts of interactions between age, environmental, and genetic factors may be associated with the development of PD [5], as well as gut microbiome dysbiosis, which is involved in the development of the disease through the microbiome-gut-brain axis [5,6,7], but they also mention a link between the disease severity and inflammatory bowel disease [8].

The growing interest in metabolic research is reflected in the increasing number of scientific reports on the subject in the last decade (Figure S1). Research is focused on the search for metabolites to define the biochemical pathways of these metabolites which can be used as potential biological markers of the disease. This might lead to a better understanding of the pathogenesis, early diagnosis, and development of disease. In the case of PD precise prognosis, early diagnosis and monitoring of disease are very important. In the present study, metabolomics and bioinformatics techniques were combined to screen for potential biomarkers of PD, using plasma samples from affected patients and healthy individuals. In order to find new potential markers, some correlations among them, and to better understand the pathophysiology, development, and disease mechanism, we used a gas chromatography-time-of-flight mass spectrometry (GC-TOFMS)-based metabolomics approach to evaluate the metabolic alterations.

## 2. Materials and Methods

### 2.1. Chemicals and Reagents

N-Methyl-N-trimethylsilyl-trifluoroacetamide (MSTFA), methoxyamine hydrochloride, and pyridine were purchased from Sigma- Aldrich Inc. (St. Louis, MO, USA). LC or GC grade methanol, isopropanol, chloroform, acetonitrile, and toluene were purchased from Merck (Darmstadt, Germany). Standard alkane series (C10-C40) and sodium sulphate were products of Sigma–Aldrich Inc. (St. Louis, MO, USA). Water was purified by a Milli-Q system (Millipore, Billerica, MA, USA).

### 2.2. Clinical Samples

The study group (PD) consisted of 25 patients with diagnosed idiopathic Parkinson’s disease. Subjects that served as controls consisted of 12 neurological patients without being confirmed by neuroimaging brain damage and extrapyramidal symptoms. Patients with severe liver disease, renal failure, malignancy, cardiac dysfunctions, autoimmune diseases, and chronic inflammatory diseases were not enrolled in the study. The plasma samples were obtained at the Department of Neurology and Stroke, Medical University of Lodz, Poland. PD patients were diagnosed according to the Movement Disorder Society Clinical Diagnostic Criteria for Parkinson’s disease (MDS-PD criteria) and the clinical condition was assessed using the Hoehn-Yahr Scale [9,10]. Blood from 37 participants was collected and immediately centrifuged to obtain plasma. Samples were checked for hemolysis, which would result in red coloring of the samples due to increased free hemoglobin concentrations. Samples that underwent hemolysis were discarded. Plasma samples were aliquoted and stored at −80 °C until GC-TOFMS analysis. According to Hoehn-Yahr (H-Y) staging in 1967 [9], there are 28% (7/25) in stage H-Y1, 16% (4/25) in stage H-Y2, 32% (8/25) in stage H-Y3, and 24% (6/25) in stage H-Y4. The mean time from the onset of the disease was 5.5 ± 4.1 years. The age range of the study participants was 35–83 years. The female/male ratios were 5/7 for the controls and 8/17 for PD. The average ages were 56 for the controls and 69 for PD. Stratification of the tested population, demographic, and clinical information are shown in Table 1. The study was approved by the Bioethics Committee of the Medical University of Lodz, Lodz, Poland (No. RNN/399/17/KE) and the work was conducted in accordance with the Declaration of Helsinki ethical guidelines. Regarding the medication, PD patients were treated using L-dopa (82%, 150–900 mg/day, mean dose 590 mg/day), amantadine (45%, 100–300 mg/day, mean dose 210 mg/day), and dopamine agonists (41%, 4–8 mg/day, mean dose 6.7 mg/day). None of them obtained monoamine oxidase B inhibitors or catechol-O-methyl transferase inhibitors.

### 2.3. Sample Preparation

The blood plasma was immediately separated and kept frozen at −80 °C for metabolomic analyses. Extraction and derivatization were performed according to the method proposed by Fiehn (2016) [11]. Plasma samples were transported on dry ice and slowly thawed on ice. After thawing, samples were vortexed for 10 s and shortly centrifuged. For extraction, to 30 µL of plasma 1 mL of ice-cold extraction solvent was added [acetonitrile:isopropanol:water (3:3:2, *v*/*v*/*v*)], vortex-mixed for 10 s and shaken for 5 min at 4 °C. The samples were then centrifuged at 13 000 rcf for 2 min at 4 °C. An amount of 450 µL of supernatant was extracted carefully and evaporated to dry at 30 °C in a rotary vacuum concentrator. All samples with repetitions, biological pools, quality control samples (QCs), and reagent blank control and method blank control were prepared and derivatized as one complete set.

### 2.4. Derivatization

To dry the metabolic extracts, 10 µL of methoxyamine hydrochloride, which was dissolved in dry pyridine at a concentration of 20 mg/mL, was added. Samples were shaken and kept at 37 °C for 30 min. Finally, the samples were trimethylsilylated with 90 µL of N-methyl-N-trimethylsilyltrifluoroacetamide (MSTFA). Silylation reaction was continued for 30 min at 37 °C. All samples were centrifuged, transferred to glass chromatographic vials, and then subjected to the GC/MS analysis.

### 2.5. Gas Chromatography-Time-of-Flight Mass Spectrometry (GC-TOFMS) Analysis

To determine metabolites in the plasma, samples were analyzed by GC-TOFMS. For the derivatized samples, 0.5 µL of aliquot was injected in the splitless mode using an autosampler into an Agilent 7890B gas chromatograph (Agilent Technologies) equipped with Rxi-5MS fused-silica capillary column of low-polarity bonded-phase (30 m length, 0.25 mm ID, and 0.25 μm film thickness) (Restek, Bellefonte, PA, USA). The injector temperature was set to 280 °C. The constant flow of 1 mL/min was set through the column, and helium was used as a carrier gas. The purge time was set to 70 s at a purge flow rate of 40 mL/min. The septum was purged with 3 mL/min. The column temperature was initially kept at 70 °C for 1 min and then increased from 70 °C to 300 °C at 12 °C/min, where it was held for 14 min. The transfer line temperature was set to 300 °C and the ion source temperature at 250 °C. Ions were generated by standard electron ionization energy of 70 eV. Masses were acquired from m/z 50 to 635 at a rate of 12 spectra/s and the acceleration voltage was turned on after a solvent delay of 336 s. The total run time was 34 min and 10 s. To ensure data quality for metabolic profiling, quality control samples were used. QCs were analyzed prior to the first sample injection, after each of the eight injections, and at the end of the experiment to ensure the repeatability of the measurements and stability of the instrument. Quality control solutions consisting of 23 highly pure standards of metabolites, blank samples, and biological pools were included within sequence to assess the condition of the system. To acquire and export data in the ANDII MS format, the ChromaTOF software platform for Pegasus BT (ver. 5.32) was applied.

### 2.6. Data Analysis

The obtained profiles were exported and transfered from the ChromaTOF software for Pegasus BT (ver. 5.32) to ChromaTOF (ver. 4.51.6.0) with the stat compare module for data processing. Automatic peak detection, deconvolution, retention index calculation, and library search were performed. To improve identification results and correct retention times (RT), retention indices (RI) were estimated, based on the analysis of standard alkane series mixture (C10-C36). To identify the compounds, the Mainlib and Fiehn libraries were used; quality filter assumed a similarity index (SI) > 700 and a retention index ±10. The unique quantification masses for each compound were defined and used to subsequently obtain accurate peak areas for statistical comparison. Unknown compounds and impurities (i.e., plasticizers, column bleeds, alkanes, siloxanes, etc.) were removed from the obtained table of data.

### 2.7. Statistics and Bioinformatics

Metabolomics data analysis was carried out in a web-based comprehensive metabolomics data processing tool, MetaboAnalyst 5.0, available at http://www.metaboanalyst.ca (accessed on 20 June 2022) [12]. The data were converted to a comma-separated value (.csv) plain text file in which the samples were listed in rows and the compounds were listed in columns. Statistical analysis was conducted after preliminary filter processing of the data. Metabolites with >50% missing values were removed from the analysis. The assumption of this approach is that most missing values are caused by low abundance metabolites, while too many missing values will cause difficulties for the downstream analysis. The remaining missing values were fitted to 1/5 of the minimum positive value of each variable in the original data. Next, data were filtered by their relative standard deviation (RSD) with a default set of the software system. Finally, data were normalized by a pooled sample from the control group, Pareto scaled, and log-transformed. In the Pareto scaling performance, the square root of the standard deviation is used as the scaling factor [13]. It aims to reduce the influence of large values without losing the important information concerning the structure of the data. Pareto scaled data are closer to the original data than the standardized data, but this depends very much on the large values in the data set. It is generally the preferred option in metabolomics because it is a good compromise between no scaling (centering) and auto scaling. After scaling, each variable retains its original range, but its average value is centered at zero.

On the first stage of the analysis, no outliers and noisy parameters were removed from the data set. Unbiased Pearson correlation analysis and principal component analysis (PCA) were initially used to provide an informative look at the metabolomic dataset structure and relationships between samples. PCA outcomes also provided outlier detection and further confirmation of the analysis by the supervised PLS-DA method (which is valuable in exploratory studies, where differences between experimental groups may be unknown or unpredictable).

Variable data were filtered to selectively remove low-quality data points from the metabolomic datasets based on the Benjamini and Hochberg false discovery rate (FDR) of > 0.05. The FDR is the expected proportion of false positive classifications (type I errors) to the number of total positive test results. As the outlier (samples and chemical variables) elimination was applied on the performed data, the remaining data set was re-normalized and re-scaled prior to each partial least squares discriminant analysis (PLS-DA). The model predictive ability was quantified in terms of the predictive squared correlation coefficient Q^2^ calculated by an internal validation procedure, leave-one-out cross-validation method (LOOCV), which allows the results of each sample for model fitting. This parameter takes values in a standardized range (<1) thus allowing trivial interpretation and an easy comparison of the different performance of fitting and predictive power of a model. We evaluated the goodness of model fit by calculating the coefficient of determination R^2^. The variable importance in the projection (VIP) of metabolite in the model was also calculated to indicate its contribution to the classification. The receiver operating characteristics curve (ROC) analysis was also carried out to identify the predictive ability of the individual metabolite.

We conducted a refinement strategy to elude over the parameterized model with rather poor discriminant properties. In this sense, we obtained the PLS-DA model based on the dataset filtered from outliers, and conducted a second PLS-DA analysis, including the important metabolites. We used variable importance for the projection (VIP) criterion that considers the contribution of a specific predictor for the explained variability in the response. Re-defined list of variables contained metabolites with a high (>1.0) VIP score for at least one of the components considered.

## 3. Results

**Figure 1:**
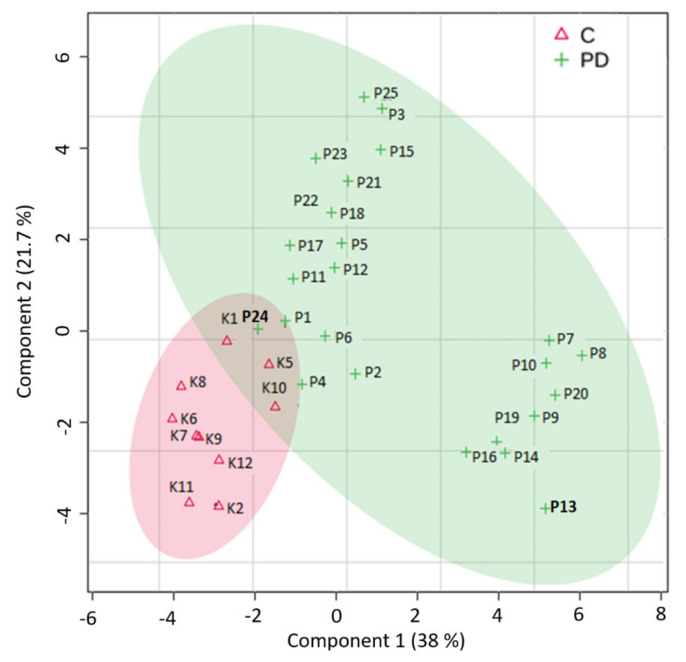
Partial least squares-discriminant analysis (PLS-DA) between normal controls and patients with Parkinson’s disease (PD). C—control group, PD—group of people with Parkinson’s disease.

To investigate the difference in plasma metabolite profiles between the PD group and controls, the data were subjected to metabolomics analysis. The partial least square discriminant analysis (PLS-DA) model was used to investigate the metabolic profiles of PD. After PLS-DA analysis, we observed a grouping of samples into two distinct groups, PD (green) and control (red), which may indicate altered or dysregulated metabolites in the plasma of PD patients compared to the control group. PLS-DA model parameters, such as determination coefficient (R^2^), accuracy, and predictive relevance (Q^2^), were 0.838, 0.914, and 0.636, respectively, for 3 components (Figure 1). To test the relevance of selected metabolites, the quality of the PLS-DA model built from them was assessed by the prediction accuracy and permutation test. The performance measures of the permutated data usually form a normal distribution, and if the performance score of the original data lies outside the distribution, the results are considered to be significant. In our analysis the cross-validation method was applied to avoid overfitting of the model and the further supervised model was confirmed by a random permutation test (*n* = 1000). PLS-DA cross-validation details were provided in Supplementary Materials (Figure S2). This result revealed good discrimination and predictive ability in this model (observed statistic *p*-value = 0.002).

**Figure 2:**
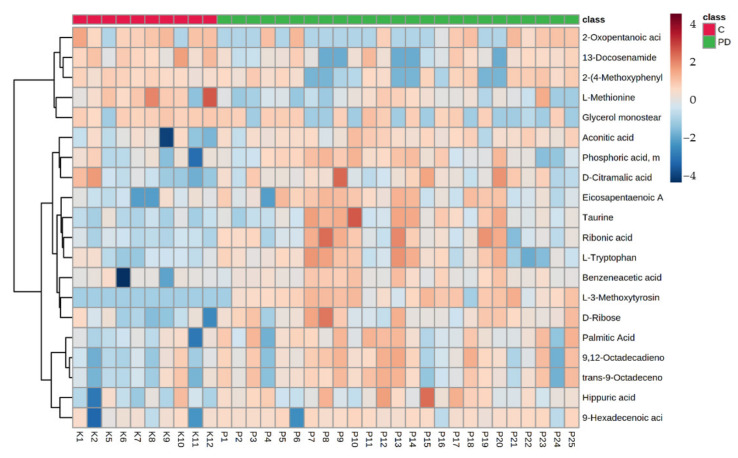
Differential metabolites and perturbed metabolic pathways in PD compared with controls. Heatmap based on Euclidean matrix distance and Ward’s clustering algorithm.

Hierarchical cluster analysis separated samples in clusters according to the metabolomics profiles (Figure 2). Heatmap shows the results validated with PLS-DA. The cell color represents the ion abundance in a plasma sample. Red indicates high abundance while blue means low abundance. The group color at the top of the figure represents the sample that belongs to the PD group (green) and the control group (red).

**Figure 3:**
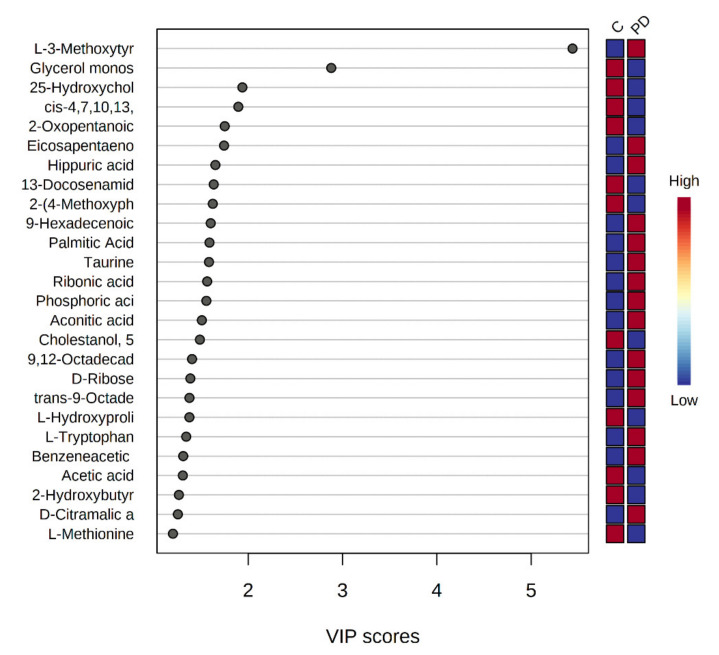
Variable importance in projection (VIP) of the metabolites in the PLS-DA model. The colored boxes indicate the relative peak height of the corresponding metabolite in each group under study. Metabolites with VIP value above 1.0 were visualized. The blue and red boxes on the right indicate whether the metabolite concentration is increased or decreased in the grouped into sets samples.

The key metabolites that contributed the most to the variance between the control samples and the PD group were selected from the application of the Variable Importance in Projection (VIP) method in the PLS-DA model (Figure 3). Among the 34 metabolites, 26 metabolites showed VIP scores >1.0, suggesting that they were the major contributing metabolites for the discrimination of the groups.

The identified metabolites (34 VIPs) were further tested for statistical significance of the difference in variance. The results are included in the Supplementary Materials (Table S1). The obtained data were analyzed using the following tests: the two-tailed Student’s *t*-test was used to test the hypothesis of equality of means of two sample groups, assuming unequal variances when data sets followed a normal distribution. Otherwise, the non-parametric Mann–Whitney U test was used. The Shapiro–Wilk test was applied to test the normality of data distribution.

In addition, fold change (FC) analysis was performed. The fold change diagram further showed that the plasma fold changes of 14 metabolites were significantly different between control and PD with a fold-change threshold Log2 > 2 (or <0.5) and *t*-test *p*-value < 0.05 (Figure S3 Supplementary Materials).

**Figure 4:**
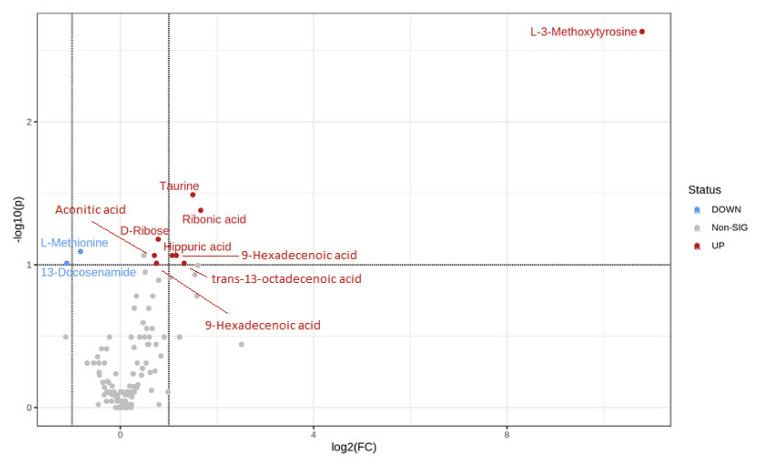
Volcano plot of the plasma metabolites of PD patients and controls, revealing 11 metabolites with a fold-change threshold Log2 > 1.5. Upregulated and downregulated metabolites are in red and blue, respectively. Nonsignificant metabolites are represented by gray dots. X-axis corresponds to log2 (Fold Change) and Y-axis to −log10 (*p*-value).

Univariate analysis via volcano plot based on fold change and adjusted *p*-value highlighted 3 metabolites (Figure 4). This made it possible to select significant features based on either biological or statistical significance. Volcano plot analysis highlighted the following metabolites: L-3-methoxytyrosine (adjusted *p*-value 0.0023, FC = 1776.4), taurine (adjusted *p*-value 0.0032, FC = 2.8), and ribonic acid (adjusted *p*-value 0.0416, FC = 3.2).

PLS-DA and variable importance in projection (VIP) scores were computed to determine how well the PD and control groups were classified by the principal components. To identify the most relevant metabolic pathways involved in PD, metabolic pathway analysis was employed to perform the pathway topology analysis. The higher VIP value indicates a stronger contribution to discrimination between the study groups. The metabolites with VIP scores > 1.0 in the partial least squares were examined and selected for their metabolic pathway analysis and then used to identify the abnormal metabolites.

**Figure 5:**
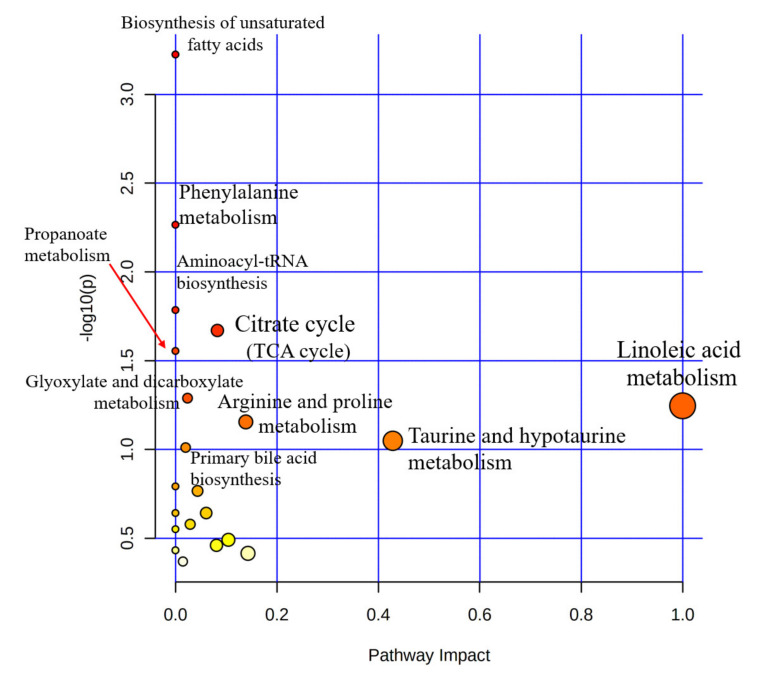
Pathway analysis of the altered metabolites in plasma.

The metabolic mechanisms among the abnormal metabolites in the plasma of PD patients were identified by pathway analysis. Metabolic pathway analysis (Figure 5) shows all metabolic pathways arranged according to the scores from the enrichment analysis (y-axis) and from the topology analysis (x-axis). Pathway analyses are conducted using identified abnormal metabolites, and the heavier the color of the pathway is, the more relevant it is to PD. Circle size and color gradient indicate the significance of the pathway ranked by *p*-value. The primary metabolic pathways closely related to PD (*p* < 0.05) are a biosynthesis of unsaturated fatty acids, phenylalanine metabolism, aminoacyl-tRNA biosynthesis, citrate cycle (TCA cycle), and propanoate metabolism. Glyoxylate and dicarboxylate metabolism were also observed.

**Figure 6:**
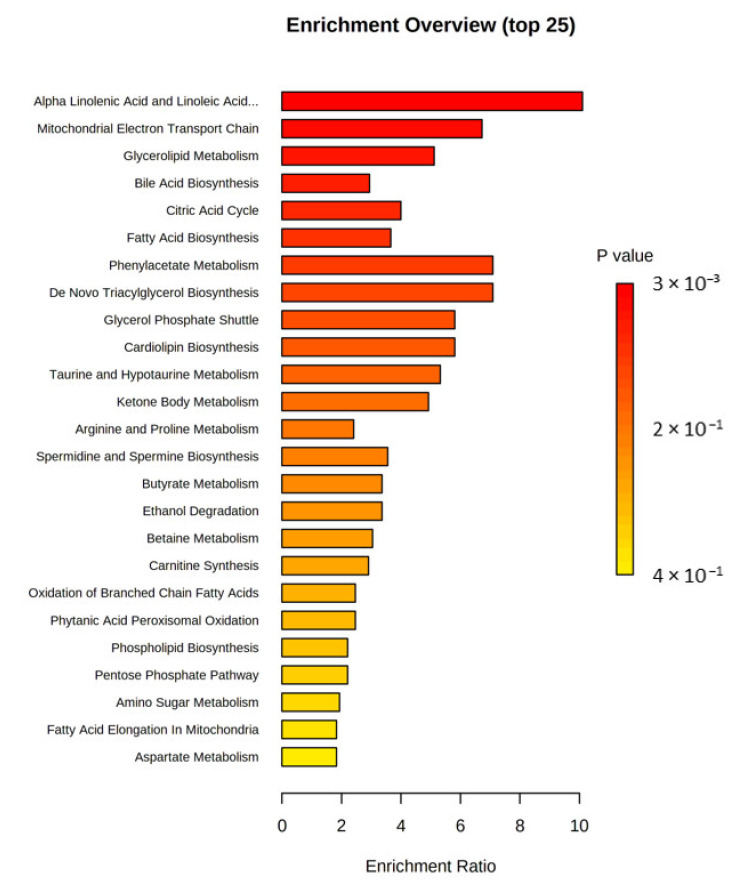
Results of the Metabolite Set Enrichment Analysis. Metabolite set enrichment analysis showed that seven differential pathways differed between the PD and control groups.

In the next step, the Metabolite Set Enrichment Analysis was applied to suggest biological pathways of potential importance (Figure 6 and Figure S4). Seven metabolite sets were found with *p*-value < 0.05, namely alpha linolenic acid and linoleic acid metabolism (3/19, expected 0.30), mitochondrial electron transport chain (2/19, expected 0.39), glycerolipid metabolism (2/25, expected 0.39), bile acid biosynthesis (3/65, expected 1.02), citric acid cycle (2/32, expected 0.50), phenylacetate metabolism (1/9, expected 0.14), and fatty acid biosynthesis (2/35, expected 0.55).

Additionally, an analysis of the receiver operating characteristics curve (ROC) was carried out to measure the ability of individual molecules to distinguish PD patients from controls. This method enables biomarker identification and performance evaluation. The areas under curve (AUC) of ROC curves were used to determine the diagnostic effectiveness of important metabolites. The AUC was 0.71–0.94 when the top 5, 10, 15, 25, 50, or 100 ions identified as significant in the *t*-test were used (Figure S5 Supplementary Materials). This result suggested that ions with *t*-test *p*-values < 0.05 as a model were a good predictor of the PD group. In a further step, ROC curve analysis for individual biomarkers to characterize the predictive value of these individual metabolites was performed independently.

Among the 14 selected metabolites (Figures S6–S9), six demonstrated an adequate potential to distinguish PD and controls, with an area under the ROC curve (AUC) greater than 0.750. L-3-methoxytyrosine showed the greatest AUC (0.957) to distinguish PD and controls, followed by aconitic acid (AUC = 0.817), L-methionine (AUC = 0.813), 13-docosenamide (AUC = 0.813), hippuric acid (AUC = 0.777), and 9,12-octadecadienoic acid (alpha-linoleic acid) (AUC = 0.757). The SVM algorithm using these six metabolites demonstrated a good ability to separate the PD group from controls (AUC: 0.975, Figure S10A). The average accuracy for 100 cross-validations was 0.92 (Figure S10B). These results support the potential of using a combination of identified metabolite biomarkers to establish a machine learning algorithm for ALS diagnosis.

## 4. Discussion

The relevance of the investigation of the plasma metabolome of PD relies on the identification of predominantly altered metabolic pathways which may lead to the discovery of possible biomarkers.

In our study, the abnormal metabolites found in PD can be divided into the following main categories: amino acids and derivatives (L-methionine (Met), L-tryptophan (Trp), L-proline (Pro), L-hydroxyproline), L-Dopa metabolite (L-3-methoxytyrosine), carboxylic acids and derivatives (aconitic acid, succinic acid), hydroxy acids and derivatives (alpha-hydroxybutyric acid), benzoic acids and derivatives (hippuric acid (HA)), fatty acids and conjugates (docosahexaenoic acid, eicosapentaenoic acid, palmitoleic acid (C16:1n-7), palmitic acid (C16:0), elaidic acid (C18:1n-9), trans-13-octadecenoic acid), lineolic acids and derivatives (alpha-linoleic acid (C18:29C)), and also other metabolites (13-docosenamide, D-ribose, taurine).

On the basis of identified metabolites, we can explore and better understand the mechanisms involved in PD if we know the correlations that occur between them. Based on multivariate and univariate statistical analyses, 1 metabolite was found relevant to L-dopa treatment. PD patients treated primarily with L-dopa were found to have elevated levels of L-3-methoxytyrosine, a major metabolite of L-dopa, whereas two metabolites, including L-3-methoxytyrosine and phenylalanine (Phe), may be related to combinational treatment [14]. In our study, two molecular pathways related to amino acids in PD patients were observed: aminoacyl-tRNA-biosynthesis (Met, Trp, Pro) and phenylalanine metabolism (Phe, hippuric acid). Amino acids associated with PD through involvement in mitochondrial metabolism are Phe, tyrosine (Tyr), and tryptophan (Trp). Phe is an essential amino acid which acts as a Tyr precursor. In contrast, Tyr and Phe are precursors of catecholamines such as dopamine, norepinephrine, epinephrine, and tyramine. Changes in the levels of large neutral amino acids such as Phe, Trp, or Tyr have been reported [14,15,16,17]. Disturbances in the levels of Tyr and Phe in patients with PD were also observed by Zhang et al. (2022). The authors pointed out the correlation of Tyr and Phe with H–Y stage and the gut microbiota, indicating that the composition of the microbiota may vary with disease progression, which consequently leads to increased plasma amino acid dysregulation [17]. Figura et al. (2018) also observed significant differences in the level of Phe, but also alanine (Ala), arginine (Arg), and threonine (Thr). In the case of all determined amino acids, there was an observed correlation between higher serum levels of amino acids with shorter disease duration and lower levels in PD patients with longer disease duration. The authors indicate that the likely mechanisms of amino acid concentration changes in PD include the effects of oxidative stress, but also the effects of mitochondrial dysfunction, altered amino acid metabolism, and malabsorption, as well as the effects of neurodegenerative processes in the brain. The use of aromatic L-amino decarboxylase inhibitors and dopaminergic drugs by patients is also not without impact [15]. Moreover, Tyr and Phe are crucial substrates for the production of the neurotransmitter dopamine, a deficiency of which is observed in PD patients [17,18,19]. In our study we observed lower levels of Met in PD patients. Mally et al. (1997) observed decreased levels of Met in the serum of PD patients [20]. The same results were presented by Meoni et al. (2022) [21]. In contrast, different results were presented by Trupp et al. (2014) [22]. Met plays an important role against oxidative stress damage. Met is involved in the oxidative stress response and is also implicated in caloric restriction phenotypes and ageing by acting as a scavenger of reactive oxygen species [23]. Abnormal Met levels may indicate altered energy metabolism in PD patients. Met is produced by the betaine–homocysteine methyltransferase from homocysteine and betaine. Postuma et al. (2004) and Rozycka et al. (2013) suggested that homocysteine accumulation in blood and cerebrospinal fluid (CSF) may be a risk factor for dementia and PD. This fact could explain the determined lower levels of the end products—Met in PD patients [21,24,25].

Another amino acid considered to be a differentiating metabolite in PD is proline (Pro). Picca et al. (2019) observed higher serum Pro levels in older PD patients compared to controls (mean age of 73.1 ± 10.2 years for PD patients (*n* = 20) and 74.6 ± 4.3 years for controls (*n* = 30)) [19]. The same results were obtained by Shao et al. (2018). They observed an increased level of Pro in plasma PD patients [14]. The increased levels of Pro, of which ornithine is a precursor, increase collagen synthesis, the accumulation of which increases the negative effects of the disease. The increase in collagen production shifts the immune system from “fight mode” to “fixing mode–wound healing” program [19].

Differences in amino acid profiles may be related to different dietary habits and, more specifically, protein intake between the compared groups. Such differences may also result from the intestinal dysbiosis present in PD [26]. In the study conducted by Pietrucci et al. (2019) 152 fecal samples were analyzed. They observed that pathways involved in amino acid metabolism (biosynthesis of Phe, Tyr, and Trp) were reduced in PD samples. They also emphasized that the alteration of the composition of the microbiota of patients with PD is associated with the pro-inflammatory environment in the gastrointestinal tract, which may interfere with the biosynthesis, absorption, and transformation of amino acids acting as precursors of neurotransmitters [27].

We observed disturbed Trp metabolism in PD patients. Gonzalez-Riano et al. (2021) reported decreased levels of Trp and their co-metabolites in PD patients [3]. Similar results were obtained by Luan et al. (2015). Urinary tryptophan catabolite levels were significantly elevated in patients with early-stage of PD [28]. Molina et al. (1997) reported a reduction in plasma Trp levels in PD patients, which is consistent with the other results presented [29]. Alterations in Trp metabolism are associated with mitochondrial dysfunction, altered brain energy metabolism involved in neurodegeneration, and psychiatric symptoms. A disturbed Trp metabolism is also associated with inflammatory activation of the kynurenine pathway in PD patients. The presence of neurotoxic kynurenine metabolites correlates with the severity of disease symptoms, hence, the conclusion that metabolites of the kynurenine pathway are involved in pathological processes in PD [30]. It has been postulated that dysregulation of Trp metabolism leads to neurotoxicity, which may act as a trigger for the development of PD [3,31].

In patients with PD, metabolic disturbances of amino acids may be related to several factors. For example, increased plasma amino acid consumption may be due to increased energy expenditure in PD patients [32]; numerous gastrointestinal dysfunctions in PD can interfere with the proper absorption of exogenous amino acids from food; deposition of α-synuclein in the gastrointestinal tract [33]; the presence of dysbiosis of the intestinal microbiota, causing disturbance in the metabolism of branched-chain and aromatic amino acids, which directly affects changes in the plasma levels of these amino acids [17,34]. The levels of Phe and Tyr are related to the daily dose of L-dopa. The higher dose of L-dopa compromises the absorption of the amino acids because they compete with the antiparkinsonian drug in utilizing the stereospecific transport system in the small intestine [35]. Metabolic changes, including fluctuations in circulating amino acids levels, may be considered as transmitters of their changes in the central nervous system [17,18,19].

The citrate cycle (TCA cycle) is an important pathway in the production of ATP. In our study, energy metabolism through the TCA cycle is one of the altered pathways and it is directly related to energy production. Energy metabolism together with other altered metabolic pathways is associated with the development of PD. In PD, an aggregation of α-Syn during the onset of the neurodegenerative processes down-regulated the metabolism of Gly, Ser, and Thr, and the TCA cycle. Such a decrease indicates insufficient energy but also mitochondrial dysfunction in PD [36]. Recently, the LC–MS metabolomics-based study has shown that in the plasma of patients with PD, altered levels of aconitic acid were observed [36]. In our research, we also observed an increased aconitic acid level in PD plasma samples, which may suggest an effect on the TCA cycle and mitochondrial dysfunction. The same results were obtained by Wu et al. (2016), who observed elevated levels of citric acid in the CSF of patients with PD [37]. In addition, elevated levels of compounds which are part of the TCA cycle, α-ketoglutarate and pyruvate, were observed by Willkommen in the CSF of PD patients [38]. Trupp et al. (2014) analyzed compounds linked to the citrate cycle intermediate (TCA cycle) from plasma and CSF. They observed an increased level of malate in PD patients [22]. Several studies showed reduced levels of TCA metabolites in postmortem brain or cell cultures. The TCA cycle and fatty acid metabolism play a significant role in energy metabolism, which is altered in PD patients. Abnormalities of the TCA cycle have been linked to PD progression and α-synuclein pathology, while fatty acid metabolism may be associated with α-synuclein aggregation [3]. Li et al. (2022) noted that altered levels of aconitic acid in PD patients begins many years before the development of the disease and are maintained throughout the course of the disease [5]. In this study, reduced levels of succinic acid were observed in plasma PD patients compared to controls. Similar observations were noted by Pathan et al. (2021). Researchers observed lower levels of succinate in samples from patients with PD. The authors indicated that increased levels of succinate suggested an effect on the TCA cycle and mitochondrial dysfunction [39]. Mitochondrial dysfunction plays an important role in the pathogenesis of PD, especially the defects in the mitochondrial respiratory chain complex-I may be responsible for neurodegeneration in PD through reduced ATP synthesis. Therefore, increased succinate levels may be associated with mitochondrial dysfunction, neurodegeneration, and PD [39]. Kumari et al. (2020) observed a positive correlation between the levels of succinate and the motor score in PD, but no correlation between urinary metabolite levels and disease duration [40]. Moreover, PD fecal samples clearly showed significantly lower levels of succinic acid, which was associated with lower PD severity and positively correlated with constipation [41].

We have found that hippuric acid (HA) was over-expressed in the plasma of patients with PD. Intestinal dysbiosis contributes to PD through signaling via microbial metabolites. Some of the most common gut metabolites are hippuric acid (HA). In the research conducted by Chen et al. (2022) in PD patients (*n* = 56), significant higher plasma levels of HA were observed compared to controls (*n* = 43). The authors indicate that aberrant gut microbial metabolites of HA and other metabolites associated with specific gut microbiota changes were observed in patients with PD, which is related to the relative abundance of proinflammatory gut bacteria [42]. HA was previously reported to be increased in the serum metabolic profile of patients with PD. Increased levels of HA may be due to gut dysbiosis and changes in metabolite production levels in PD patients. HA is produced by the conjugation of benzoic acid with glycine. This reaction occurs in the liver, but also directly in the intestine and kidney. It should be noted that HA levels increase when larger amounts of phenylalanine-rich foods are provided or when this amino acid is subjected to the direct action of intestinal bacteria [43]. Moreover, HA has also been linked to an altered odor in sebum PD patients. This may indicate altered microbial activity on the skin of PD patients, suggesting altered skin microflora and skin physiology, causing changes in the production of metabolites such as HA [44].

Alpha-hydroxybutyrate (a-HB) is an organic acid which is derived from alpha-ketobutyrate (a-KB). During the formation of a-KB in a reaction catalyzed by lactate dehydrogenase (LDH) or alpha-hydroxybutyrate dehydrogenase (a-HBDH), an isoform of LDH present in the heart, a-HB is formed as a by-product. Accumulation of a-HB is thought to occur in vivo when the formation of a-KB exceeds its rate of catabolism leading to substrate deposition, or there is a reduction in the rate of the dehydrogenase that catalyzes the conversion of a-KB to propionyl-CoA [45]. Alpha-hydroxybutyric acid is mainly produced in the liver, where glutathione synthesis or L-threonine catabolism takes place [46]. Our results showed decreased levels of alpha-hydroxybutyric acid in PD patients. The same results were obtained by Pathan et al. (2021). They observed increased plasma levels of a-HB in PD patients compared to controls [39]. Hence, the conclusion that these changes may indicate increased activity of the glutathione pathway [46].

Our study showed a significant dysregulation in the biosynthesis of unsaturated fatty acids (FFAs), especially the metabolism of linoleic, docosahexaenoic acid (DHA), and eicosapentaenoic acid (EPA) in PD patients, which may be involved in the etiopathogenesis of disease. FFAs are needed to form membranes, generate signaling molecules, and provide energy for beta-oxidation. Recent studies revealed that the interaction between FFAs and monomeric α-synuclein accelerates the production of α-Synuclein assemblies [47]. One of the monounsaturated fatty acids is palmitoleic acid (16:1n-7), which is a significant compound for human metabolism. It exhibits anti-inflammatory properties with a protective effect on neurons. Palmitoleic acid is one of the principal components of the human adipose tissue, muscle, and liver together with oleic (18:1n-9), linoleic (C18:2), palmitic (C16:0), and stearic (C18:0) acids. In patients with PD, we observed a significant alteration in the levels of these compounds. Palmitoleate has been associated with decreased lipid accumulation in the liver and increased insulin sensitivity. Moreover, an increasing circulating serum of palmitoleate influences adipose tissue abundance [48].

Shao et al. (2021) also observed in the plasma of PD patients a metabolic dysregulation in the biosynthesis of unsaturated FFAs (palmitic acid, linoleic, linolenic, and arachidonic acids) [14]. Gonzalez-Riano et al. (2021) suggested that alteration in the levels of stearic, palmitic, palmitoleic, oleic, linoleic, and arachidonic acids can be a significant metabolic marker for the potential early detection of PD [3]. Trupp et al. (2014) detected decreased levels of palmitic acid and linoleic acid in PD. Higher levels of amino acids and lower levels of several similar C16 and C18 saturated and unsaturated fatty acids may suggest the presence of significant changes in pathways of energy usage in PD [22].

Additionally, other abnormal metabolites we found in PD can be categorized as compounds from the bile acid biosynthetic pathway (taurine), pentose phosphate pathway (D-ribose), and others (13-docoseamide). Taurine exhibits inhibitory properties as a neurotransmitter. It is produced and secreted by neurons during stress, as well as in mitochondrial dysfunction. By regulating calcium influx and stimulating antioxidant gene expression, taurine increases neuronal survival [49]. The increase in taurine levels in the mouse brain after α-synuclein injection indicates the crucial importance of sulfur metabolism, primarily taurine in the CNS [50]. Hertel et al. (2019) suggested that sulfur metabolism is altered in PD in interaction with gut microbiota. Moreover, these changes may translate via taurine-conjugated bile acids into variability in PD severity of clinical symptoms [49]. According to Kumari et al. (2020) increased concentrations of taurine were observed in PD saliva as compared to controls [51]. NMR-based metabolomics studies identify taurine in Alzheimer’s disease (AD) as a potential biomarker.

Recent studies show dysregulation of the pentose phosphate pathway (PPP), which includes D-ribose, in PD [52,53]. PPP does not provide energy supply but coordinates anabolic biosynthesis and redox homeostasis by controlling the intracellular products ribose-5-phosphate and NADPH. Brains and immune cells display high activity of glucose-6 phosphate dehydrogenase (G6PD). G6PD causes PPP acceleration. The study reveals dysregulation of G6PD enzymes in brains of PD patients, which is mediated in chronic dopaminergic neurodegeneration and locomotor impairment. The pathogenic roles of G6PD and PPP in PD is not well understood and requires further research [54].

There are very few reports of 13-docosenamide in the available literature. Information can be found that this compound has been identified in human blood [55]. Furthermore, 13-docosenamide is not a naturally occurring metabolite. This metabolite is found in people exposed to this compound or its derivatives, with agents from environmental and occupational sources. It has also been identified in benign prostatic hyperplasia model mice. Serum metabolite profiles of mice were analyzed and 3 potential biomarkers were discovered and identified to distinguish between the study groups. One of these biomarkers was 13-docosenamide. As suggested by the authors, the presence of this compound indicated that the occurrence of benign prostatic hyperplasia is closely related to abnormalities in lipid metabolism [56].

This study had, however, important limitations such as the limited sample size and unequal groups in terms of gender. The unequal group size was due to the need to exclude some samples from the control group because of the observed hemolysis of the blood sample, which results in red coloring of the samples. Secondly, patients in the PD group were slightly older than those in the control group. Thirdly, a slightly higher proportion of patients with PD at an advanced stage of the disease was observed (H–Y ≥ 3), which may conceal the alterations of metabolites in those at an early stage (H–Y ≤ 2). Additionally, the power analysis may not be sufficient to detect smaller changes of metabolites in the studied groups. Furthermore, the diet and lifestyle of the patients participating in the study may have influenced the results obtained. Finally, our analysis may have been influenced by drug interactions as well as other random and unmeasured factors, which may have also contributed to the metabolic differences between the groups. Nevertheless, our study clearly captures the key metabolic features observed in the plasma of PD patients. These metabolic changes provide more potential opportunities to study pathogenesis, monitor clinical progression, and observe the efficacy of the applied treatment in PD patients. It is still unclear whether the discussed metabolites are specific to PD. Future analysis of a larger population, comparison with serum or CSF of patients, as well as carrying out questionnaires on diet and lifestyle may lead to important findings.

## 5. Conclusions

GC-TOFMS analysis and multivariate statistical analysis allowed the identification of a distinct plasma metabotype in patients with PD in contrast to controls. This metabolomic analysis demonstrates new plasma biomarker candidates for PD, including L-3-methoxytyrosine, aconitic acid, L-methionine, 13-docosenamide, hippuric acid, and 9,12-octadecadienoic acid. Our findings point towards an association between metabolic dyshomeostasis and several impaired metabolic pathways, including amino acid and TCA cycle metabolism, alpha linolenic acid and linoleic acid metabolism, mitochondrial dysfunction, bile acid biosynthetic pathway, and pentose phosphate pathway in patients with PD compared to controls. Due to the complexity of PD, the development of symptoms and progression of the disease is caused by several interrelated metabolic pathways. Future research on a larger population of individuals and a group of PD patients more diverse in terms of disease severity, in order to precisely quantify these biomarkers, determine their role in pathophysiology, monitor the disease, and evaluate and possibly modify the treatment used are warranted.

## Figures and Tables

**Table 1:** Stratification of tested population (mean ± SD).

Study Population	PD	Control
Participants	25	12
Gender		
Female	8	5
Male	17	7
Age (years)	68.7 ± 7.0	56.2 ± 18.3
BMI	26.7 ± 3.6	30.5 ± 9.1
Disease duration (years)	5.5 ± 4.1	-
Hoehn and Yahr scale	2.4 ± 1.1	-
H–Y1	7 (28%)	-
H–Y2	4 (16%)	
H–Y3	8 (32%)	
H–Y4	6 (24%)	

## Data Availability

The datasets generated during the current study are available from the corresponding author on reasonable request.

## Supplementary Materials

The following supporting information can be downloaded at: https://www.mdpi.com/article/10.3390/biomedicines10123005/s1, Figure S1: Number of scientific reports from the last ten years (2012–2021) in the PubMed database according to the specified queries (data as of 7 November 2022); A—the keywords used to search the database (“metabolomics” and “Parkinson’s disease”), (“body fluids” and “PD” and „metabolomic”); B—the keywords used to search the database (“metabolomics” and “PD”); Figure S2: Evaluation of the PLS-DA model. (A) A leave-one-out cross-validation (LOOCV) was performed. The class discrimination performance was measured using classification accuracy (blue bars) explained variation parameter R2, (pink bars), that indicate the goodness of fit, and a measure of predictive validity of the model Q2 (light-blue bars). The highest predictive relevance (Q2 value) is marked by *. (B) PLS-DA model parameters for 3 components: R2 = 0.838, Q2 = 0.636. (C) Permutation test of the PLS-DA model (n = 1000), indicating suitable model without overfitting; Figure S3: Fold change diagram shows fold change of different metabolites; Figure S4: Network summary from an over-representation analysis (ORA). The nodes represent metabolite sets enriched in the data; Figure S5: The receiver operating characteristic curve (ROC curve) analysis for the composite metabolites; Figure S6: ROC curves analysis for the 4 significantly changed metabolites (L-3-Methoxytyrosine, Aconitic acid, Hippuric acid, L-Methionine); Figure S7: ROC curves analysis for the 4 significantly changed metabolites (13-Docosenamide, D-Ribose, 9,12-Octadecadienoic acid, N-Acetyl-D-glucosamine); Figure S8: ROC curves analysis for the 4 significantly changed metabolites (trans-9-Octadecenoic acid, Heptadecanoic acid, Palmitic acid, Benzeneacetic acid); Figure S9: ROC curves analysis for Stearic acid; Figure S10: Diagnosis of Parkinson’s disease by identified metabolites. (A) Receiver operating characteristic analysis using 6 metabolites with an area under the ROC curve (AUC) greater than 0.750 such as L-3-methoxytyrosine, aconitic acid, L-methionine, 13-docosenamide, hippuric acid, 9,12-octadecadienoic acid. One-hundred-fold cross-validations were performed, and the results were averaged to generate the plot. The 95% confidence intervals are indicated as the blue shaded area. (B) Predictive accuracy of cross-validations. The average accuracy was 0.92. CI: Confidence Interval. Reference [57] is cited in the Supplementary Materials.
