# Supplementary material for: Plasma Metabolic Disturbances in Parkinson’s Disease Patients
Source: Biomedicines. 2022 Nov 22;10(12):3005. doi: 10.3390/biomedicines10123005 (PMC9775245; doi:10.3390/biomedicines10123005)
Supplement: Supplementary file 1 [file biomedicines-10-03005-s001.zip › Supplementary Materials/Supplementary Materials.pdf]

Supplementary Materials

Metabolomic studies in Parkinson's disease

The growing interest in metabolic research is reflected in the increasing number of scientific reports on the subject in the last decade (**Figure S1**). In the literature review, there is a lot of evidence of application of the analytical techniques which are currently used in metabolomics in the studies focusing on PD. The recent increase in the number of published studies in this field indicates interest in the metabolomics techniques and PD. **Figure S1** presents a number of scientific reports according to the PubMed database using the following keywords in searching the database, sorted by best match: ("metabolomics" and "Parkinson's disease"), ("body fluids" and "PD" and „metabolomic"), and ("metabolomics" and "PD").

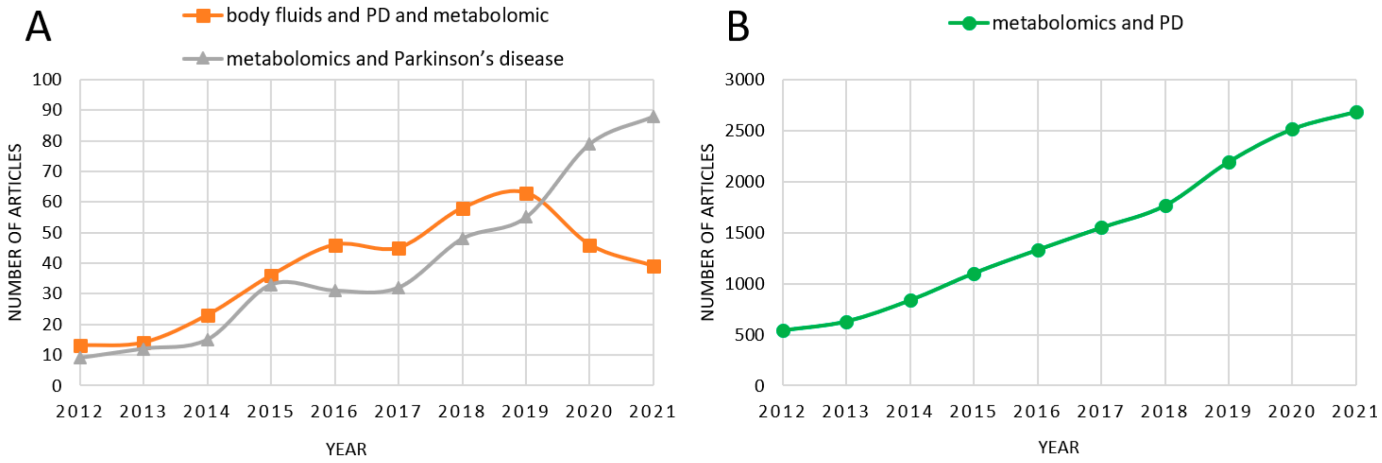

Figure S1. Number of scientific reports from the last ten years (2012-2021) in the PubMed database according to the specified queries (data as of 7 November 2022); (**A**) - the keywords used to search the database ("metabolomics" and "Parkinson's disease"), ("body fluids" and "PD" and „metabolomic"); (**B**) - the keywords used to search the database ("metabolomics" and "PD").

Evaluation of the PLS-DA model

Class separation between groups (PD and controls) was achieved by PLS-DA. A permutation test was performed to assess the significance of class discrimination.

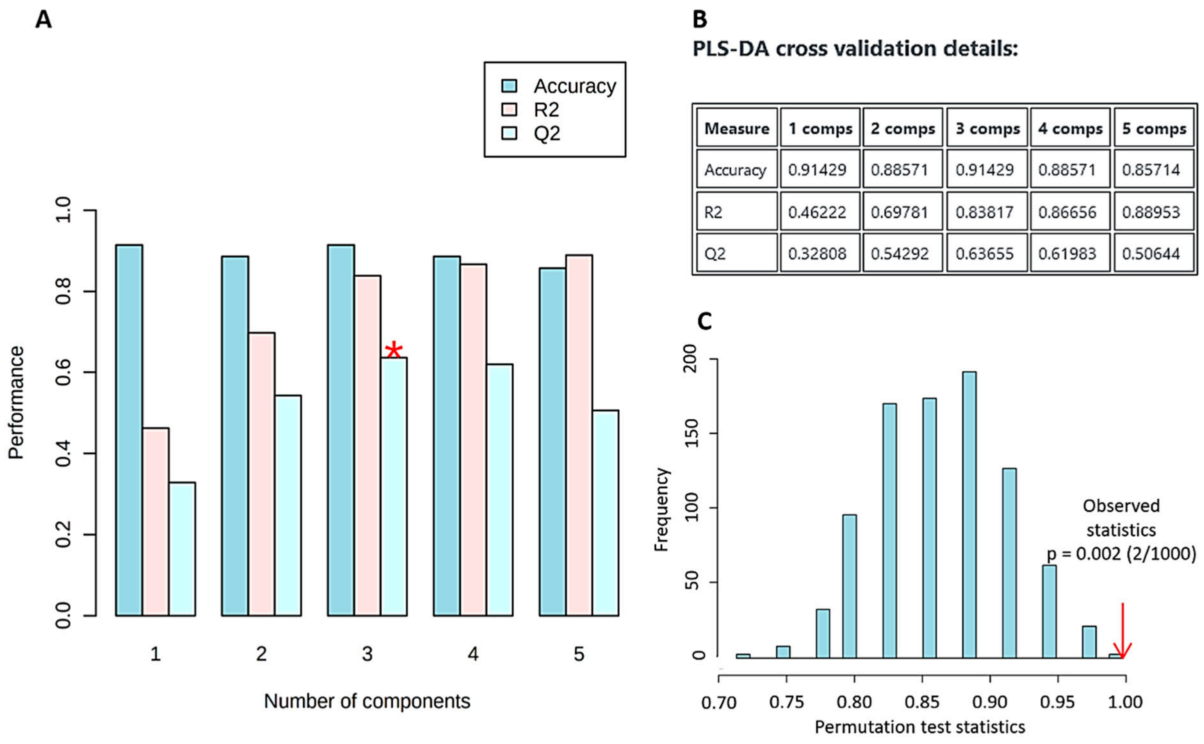

PLS-DA cross validation details:

Measure	1 comps	2 comps	3 comps	4 comps	5 comps
Accuracy	0.91429	0.88571	0.91429	0.88571	0.85714
R2	0.46222	0.69781	0.83817	0.86656	0.88953
Q2	0.32808	0.54292	0.63655	0.61983	0.50644

Figure S2. Evaluation of the PLS-DA model. (**A**) A leave-one-out cross-validation (LOOCV) was performed. The class discrimination performance was measured using classification accuracy (blue bars) explained variation parameter R2, (pink bars), that indicate the goodness of fit, and a measure of predictive validity of the model Q2 (light-blue bars). The highest predictive relevance (Q2 value) is marked by *. (**B**) PLS-DA model parameters for 3 components: R2=0.838, Q2=0.636. (**C**) Permutation test of the PLS-DA model (n=1000), indicating suitable model without overfitting.

Fold change (FC)

Fold change (FC) analysis of the metabolite abundance between PD and healthy plasma samples. The fold change is calculated as the log transformation of the ratio between the mean metabolite abundance in the PD cohort to healthy control cohort (PD/C). FC analysis has been carried out to describe the degree of quantity change between group means. 14 metabolites were significantly different between PD and control with a fold-change threshold Log2 > 2 (or < 0.5) and t-test *p*-value < 0.05 (**Figure S3**). The fold change diagram showed that 12 kinds of plasma differential metabolites were significantly increased, and 2 kinds were significantly decreased in the PD group. The levels of 2-oxopentanoic acid and 13-docosenamide were significantly downregulated in the PD group compared to controls. PD metabolism was dysregulated with increased plasma L-3-methoxytyrosine, glycerol-3-phosphate, ribonic acid, eicosapentaenoic acid, palmitic acid, L-tryptophan, taurine, trans-13-octadecenoic acid, phosphoric acid, monomethyl ester, hippuric acid, 9,12-octadecadienoic acid and also trans-9-octadecenoic acid.

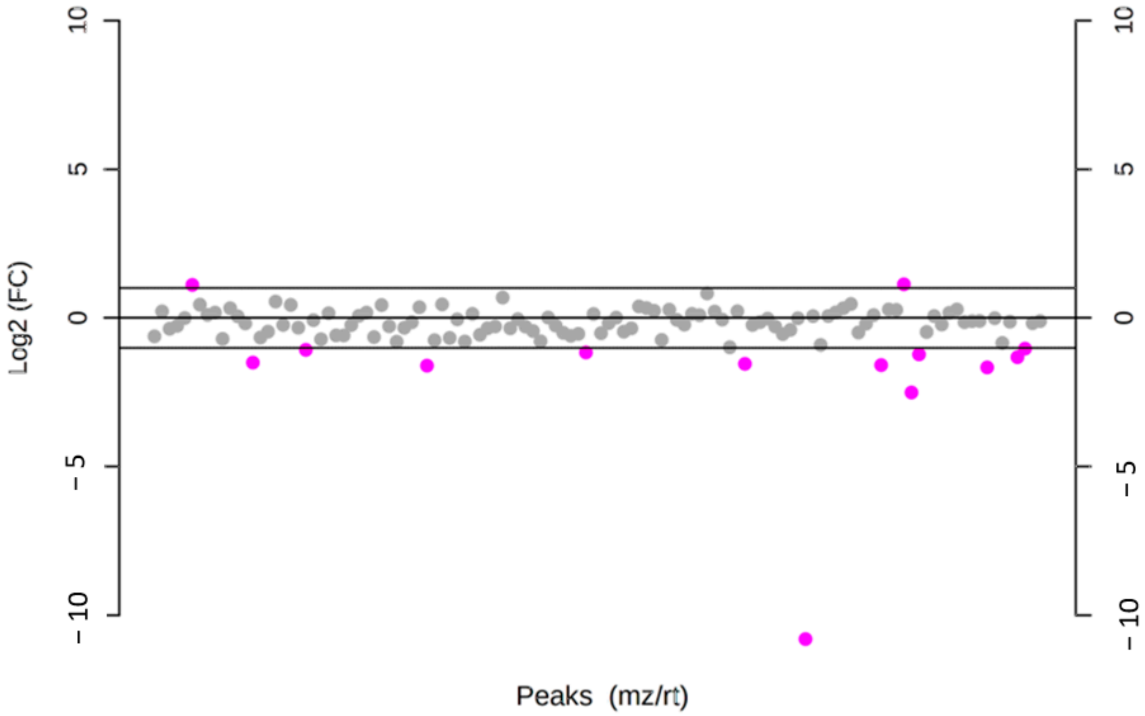

Figure S3. Fold change diagram shows fold change of different metabolites. Pink dots represent statistically significant metabolites with a p-value < 0.05 and fold-change threshold Log2 > 2 (or < 0.5).

Figure S4. shows the enrichment network where metabolite set is represented by node, with its color corresponding to its p-value in the enrichment analysis and its size corresponding to its fold enrichment. When the number of common metabolites between metabolite sets is greater than 20%, such sets are connected by a line. From the network, it is apparent that many enriched pathways, including alanine metabolism, ammonia recycling, glycine and serine

metabolism, methionine metabolism, spermidine and spermine biosynthesis, and citric acid cycle, are highly interconnected.

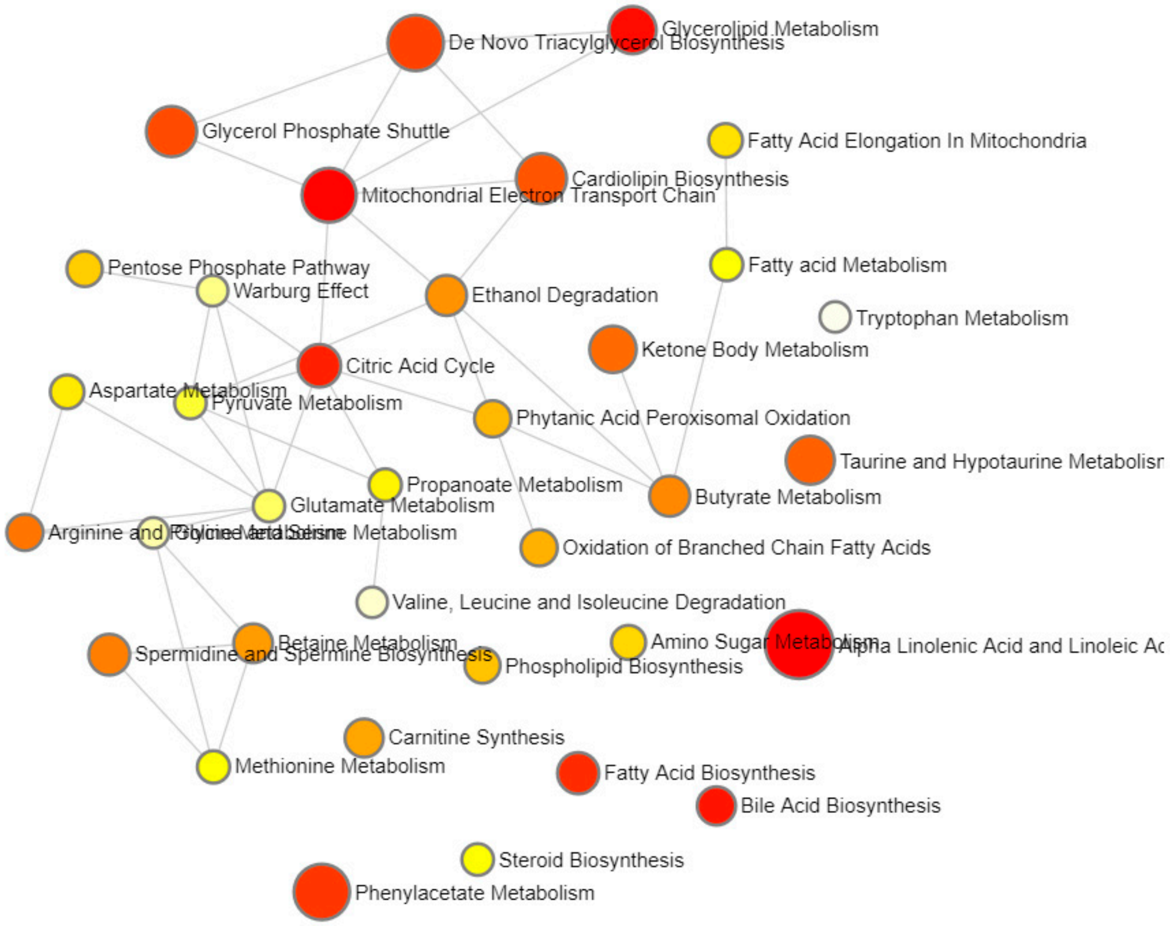

Figure S4. Network summary from an over-representation analysis (ORA). The nodes represent metabolite sets enriched in the data.

ROC analysis

A ROC curve was performed using t-test *p*-value lower than 0.05. The area under the ROC curve (AUC values) (**Figure S5**) was above 0.71-0.94 we used the top 5 or 10, 15, 25, 50, 100 t-test ions to do the biomarker analysis. Higher AUC values were when we used the top 10 t-test ions. The AUC results were considered good for AUC values between 0.7-0.8, and very good for AUC values between 0.8-0.9. Our result suggested that these groups of ions with t-test *p*-values lower than 0.05 as a model were a good/very good predictor to differ PD and control groups. The expert scale for AUC values shows quality of the test [57].

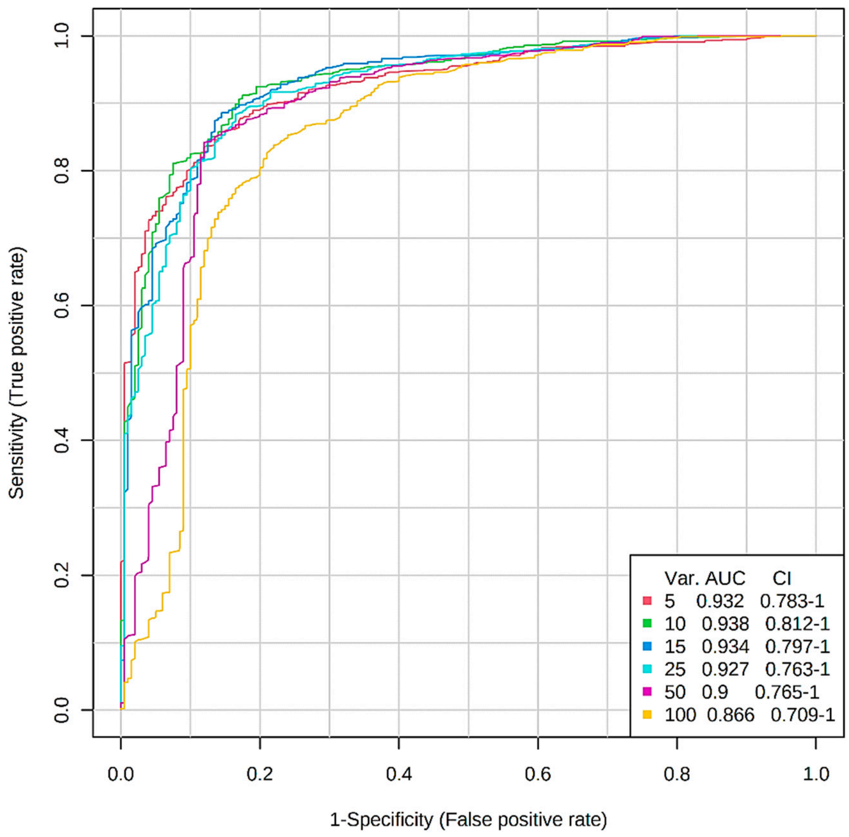

Figure S5. The receiver operating characteristic curve (ROC curve) analysis for the composite metabolites.

Receiver operating characteristic (ROC) curve (left panel) and box plot analysis (right panel) of plasma levels of metabolites. The area under the ROC curve (AUC) is in shadow. The black center line in the box plot denotes the median, the green or red boxes contain the 25th to 75th percentiles for patients with PD or controls, respectively. The black whiskers mark the 5th and 95th percentiles, and mean values are marked with yellow dots.

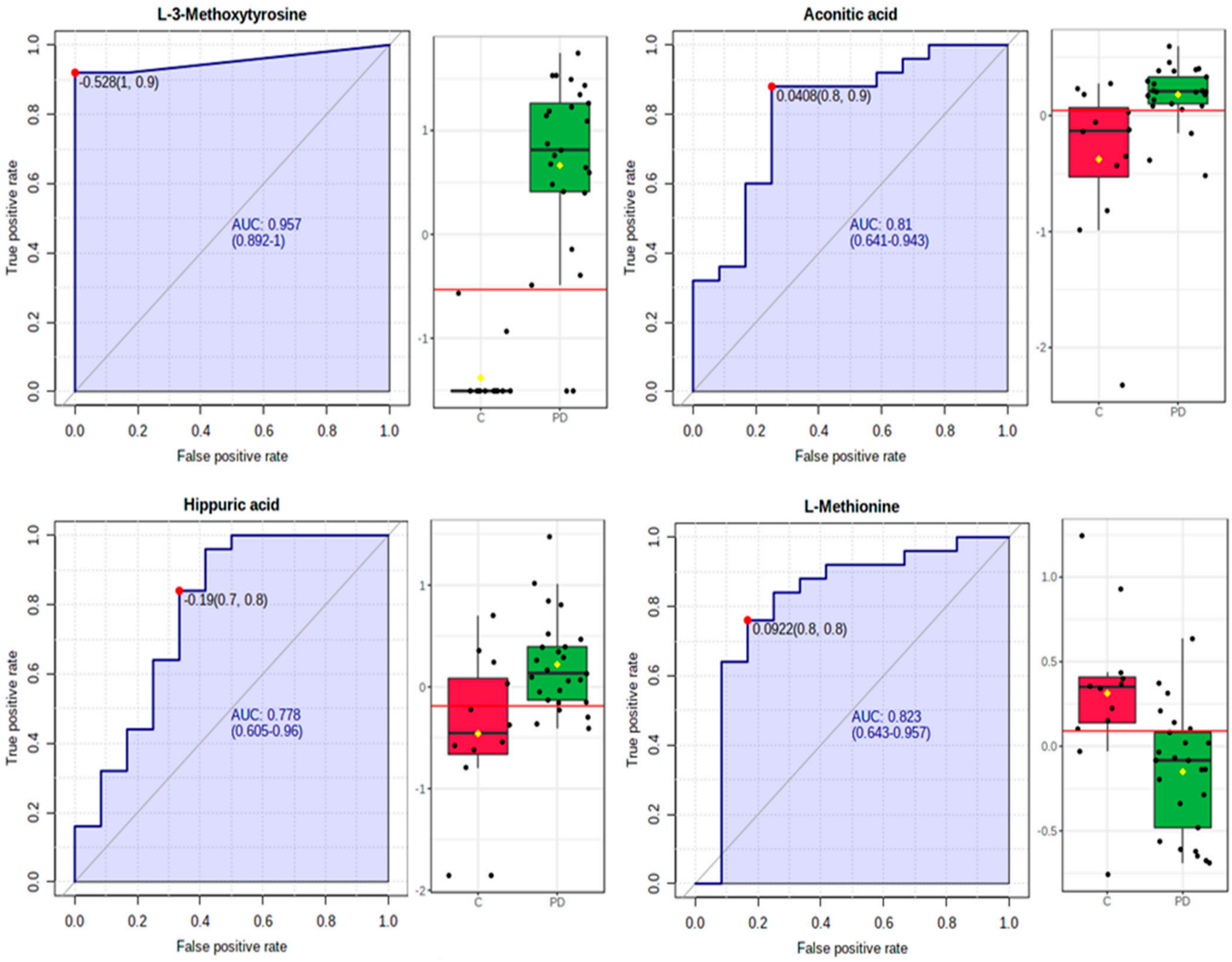

Figure S6. ROC curves analysis for the 4 significantly changed metabolites (L-3-methoxytyrosine, aconitic acid, hippuric acid, L-methionine).

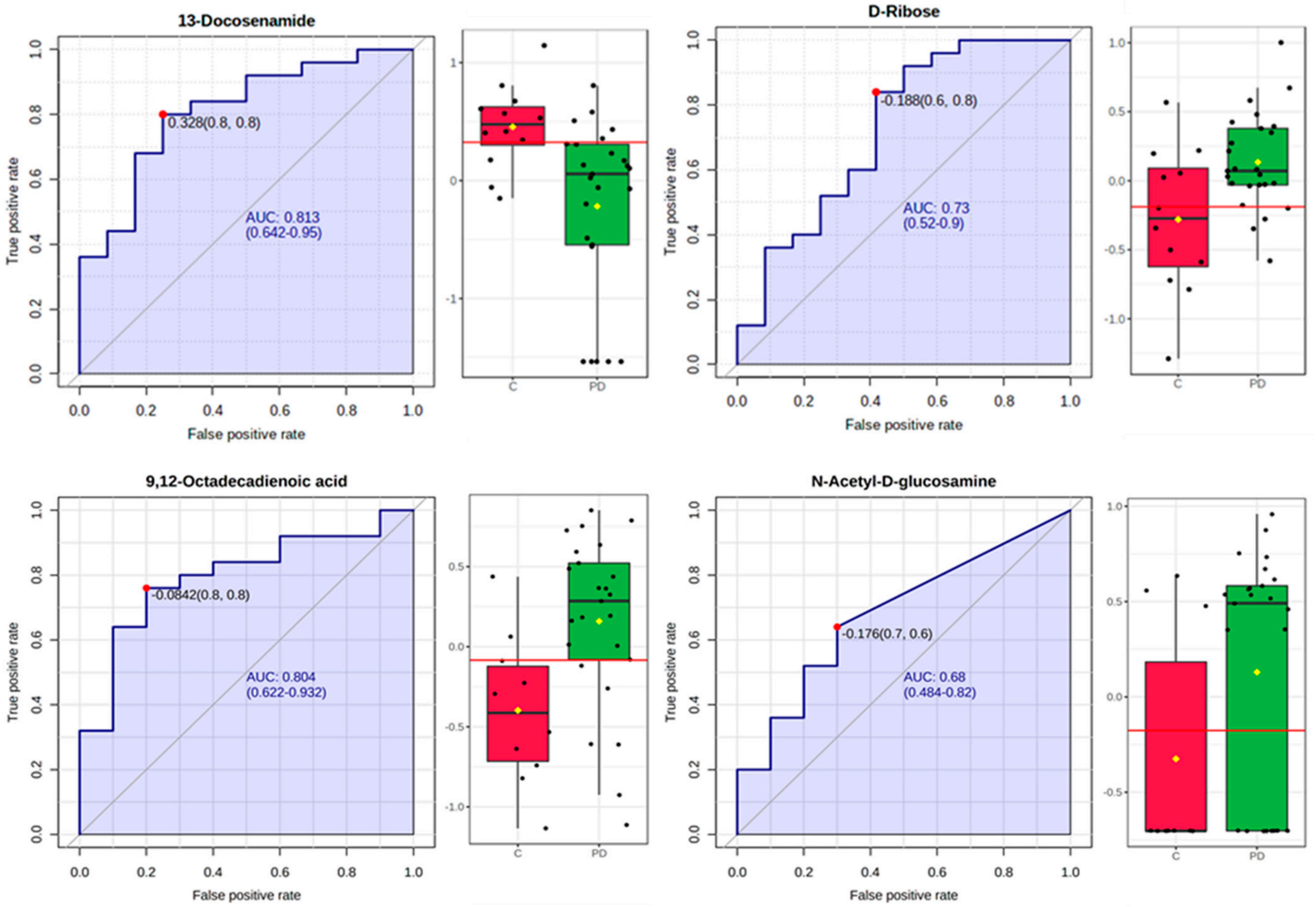

Figure S7. ROC curves analysis for the 4 significantly changed metabolites (13-docosenamide, D-ribose, 9,12-octadecadienoic acid, N-acetyl-D-glucosamine).

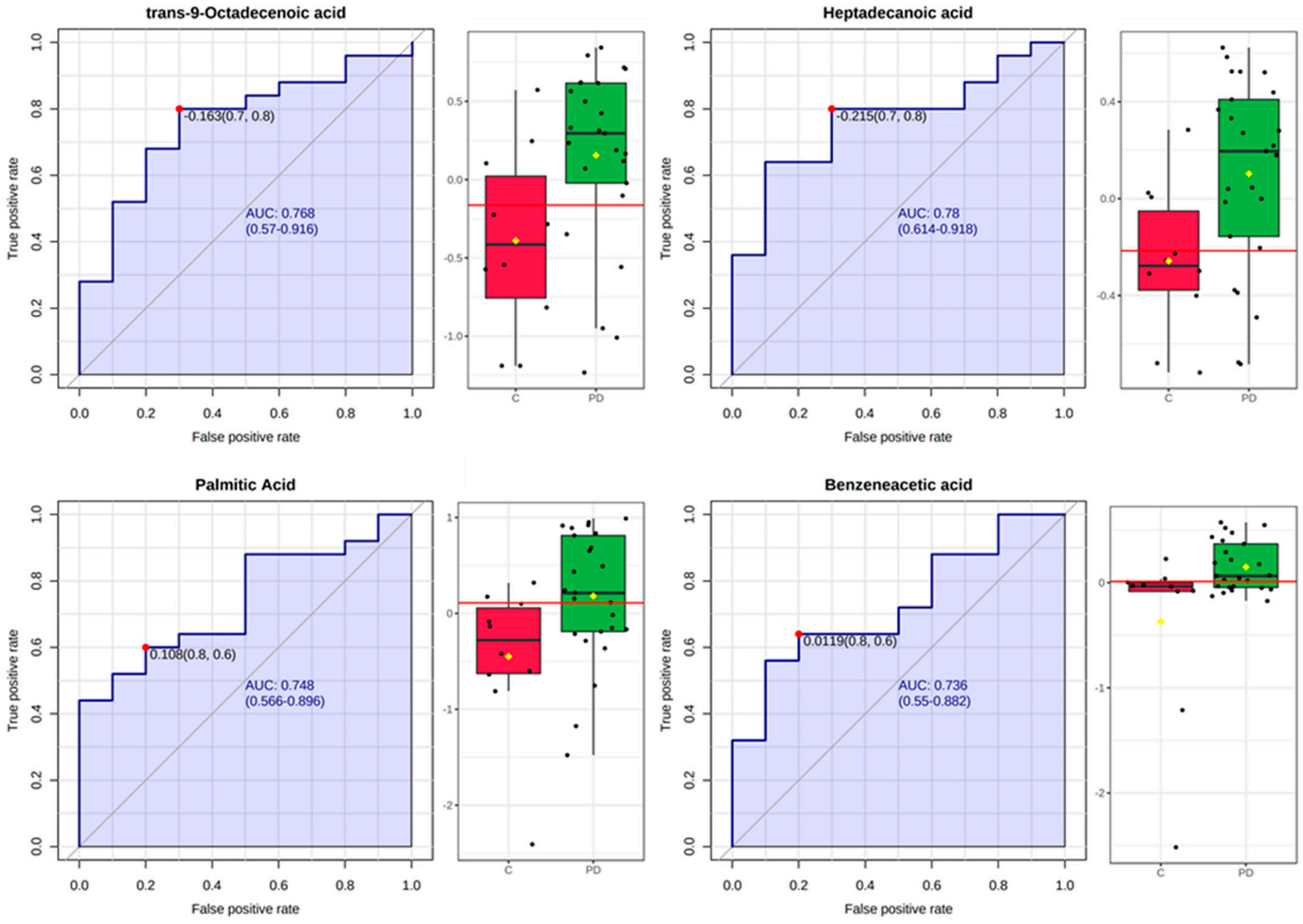

Figure S8. ROC curves analysis for the 4 significantly changed metabolites (trans-9-cctadecenoic acid, heptadecanoic acid, palmitic acid, benzeneacetic acid).

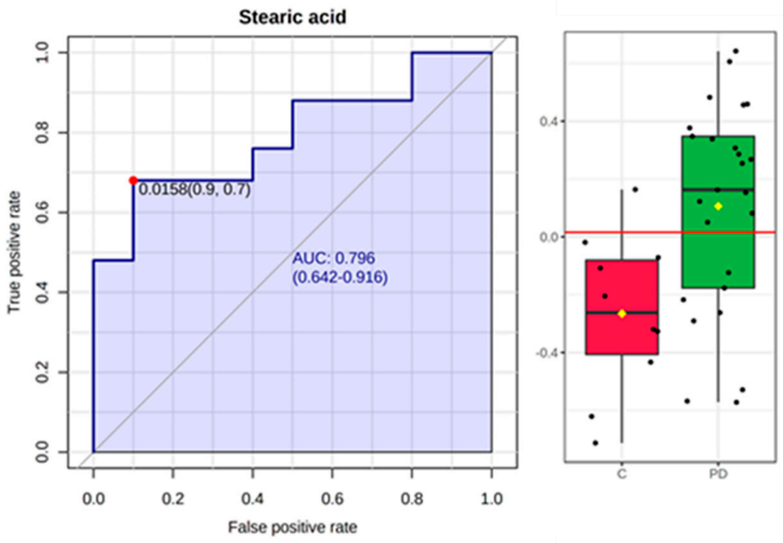

Figure S9. ROC curves analysis for stearic acid.

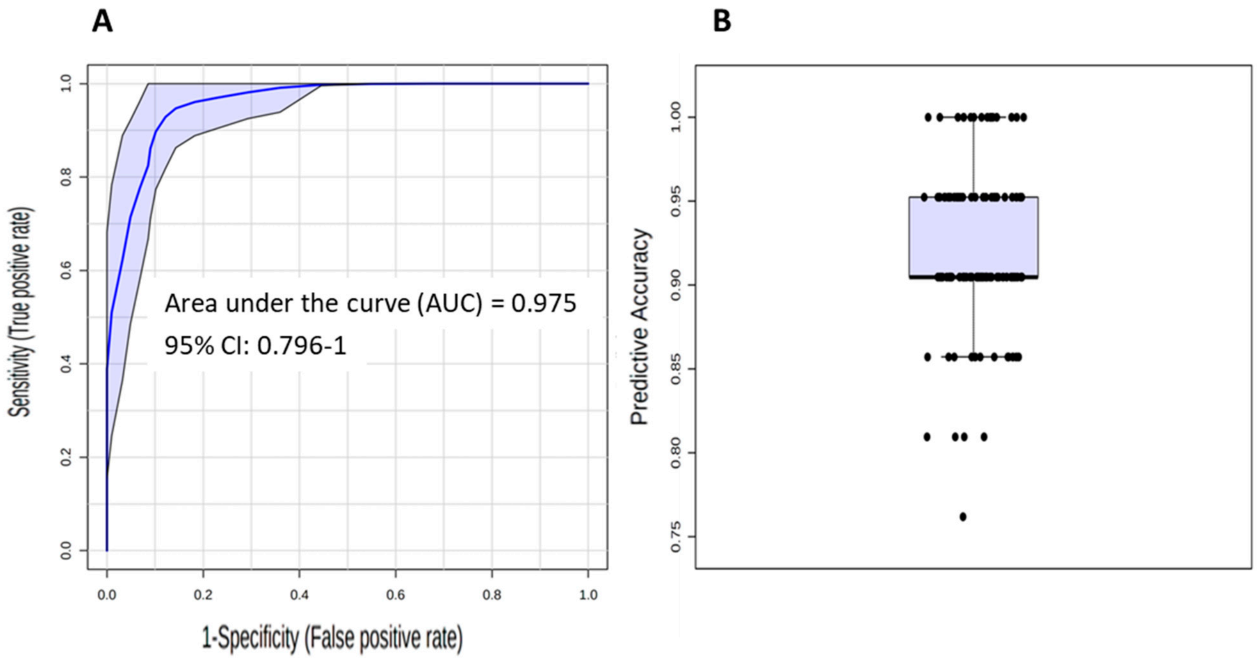

Figure S10. Diagnosis of Parkinson's disease by identified metabolites. (**A**) Receiver operating characteristic analysis using 6 metabolites with an area under the ROC curve (AUC) greater than 0.750 such as L-3-methoxytyrosine, aconitic acid, L-methionine, 13-docosenamide, hippuric acid, 9,12-octadecadienoic acid. One-hundred-fold cross-validations were performed, and the results were averaged to generate the plot. The 95% confidence intervals are indicated as the blue shaded area. (**B**) Predictive accuracy of cross-validations. The average accuracy was 0.92. CI: Confidence Interval.
